# Phenonizer: A Fine-Grained Phenotypic Named Entity Recognizer for Chinese Clinical Texts

**DOI:** 10.1155/2022/3524090

**Published:** 2022-03-23

**Authors:** Qunsheng Zou, Kuo Yang, Zixin Shu, Kai Chang, Qiguang Zheng, Yi Zheng, Kezhi Lu, Ning Xu, Haoyu Tian, Xiaomeng Li, Yuxia Yang, Yana Zhou, Haibin Yu, Xiaoping Zhang, Jianan Xia, Qiang Zhu, Josiah Poon, Simon Poon, Runshun Zhang, Xiaodong Li, Xuezhong Zhou

**Affiliations:** ^1^Institute of Medical Intelligence, School of Computer and Information Technology, Beijing Jiaotong University, Beijing 100044, China; ^2^The First Affiliated Hospital of Henan University of Chinese Medicine, Zhengzhou 45000, China; ^3^Hubei Provincial Hospital of Traditional Chinese Medicine, Wuhan 430061, China; ^4^Data Centre of Traditional Chinese Medicine, China Academy of Chinese Medical Science, Beijing 100700, China; ^5^School of Information Technologies, The University of Sydney, Sydney, Australia, Analytic and Clinical Cooperative Laboratory for Integrative Medicine, USYD & CUHK, Sydney 2006, Australia; ^6^Guang'anmen Hospital, China Academy of Chinese Medical Science, Beijing 100053, China; ^7^Institute of Liver Disease, Hubei Provincial Academy of Traditional Chinese Medicine, Wuhan 430061, China

## Abstract

Biomedical named entity recognition (BioNER) from clinical texts is a fundamental task for clinical data analysis due to the availability of large volume of electronic medical record data, which are mostly in free text format, in real-world clinical settings. Clinical text data incorporates significant phenotypic medical entities (e.g., symptoms, diseases, and laboratory indexes), which could be used for profiling the clinical characteristics of patients in specific disease conditions (e.g., Coronavirus Disease 2019 (COVID-19)). However, general BioNER approaches mostly rely on coarse-grained annotations of phenotypic entities in benchmark text dataset. Owing to the numerous negation expressions of phenotypic entities (e.g., “no fever,” “no cough,” and “no hypertension”) in clinical texts, this could not feed the subsequent data analysis process with well-prepared structured clinical data. In this paper, we developed Human-machine Cooperative Phenotypic Spectrum Annotation System (http://www.tcmai.org/login, HCPSAS) and constructed a fine-grained Chinese clinical corpus. Thereafter, we proposed a phenotypic named entity recognizer: Phenonizer, which utilized BERT to capture character-level global contextual representation, extracted local contextual features combined with bidirectional long short-term memory, and finally obtained the optimal label sequences through conditional random field. The results on COVID-19 dataset show that Phenonizer outperforms those methods based on Word2Vec with an F1-score of 0.896. By comparing character embeddings from different data, it is found that character embeddings trained by clinical corpora can improve *F*-score by 0.0103. In addition, we evaluated Phenonizer on two kinds of granular datasets and proved that fine-grained dataset can boost methods' F1-score slightly by about 0.005. Furthermore, the fine-grained dataset enables methods to distinguish between negated symptoms and presented symptoms. Finally, we tested the generalization performance of Phenonizer, achieving a superior F1-score of 0.8389. In summary, together with fine-grained annotated benchmark dataset, Phenonizer proposes a feasible approach to effectively extract symptom information from Chinese clinical texts with acceptable performance.

## 1. Introduction

The natural language processing (NLP) and information extraction (IE) techniques are the vital parts of data mining and knowledge discovery in EMRs and have become a hot research field in biomedical informatics [1, 2]. Biomedical named entity recognition (BioNER) as a basic task in biomedical IE has received constant research attention over the recent years [3–5].

BioNER is a critical task designed to identify and classify clinical terms in EMRs, such as symptoms, diseases, body parts, operations, and drugs [6]. In EMRs, the same word can refer to more than one kind of entities, while various words can describe the same entities [7]. Moreover, there are abbreviations or acronyms and multiple variants of same entities in EMRs [8]. These entities rarely or even do not occur in EMRs, but it is still a problem that cannot be ignored. Both of these problems make BioNER a challenging task. The previous BioNER methods were mainly developed for English texts. In recent years, with the Chinese medical information system gained in popularity, BioNER in Chinese clinical texts has also received extensive attention. Due to the lack of Chinese word boundaries and complexity of the form of Chinese, BioNER in Chinese texts is more difficult than that in English texts [9].

As an emergent infectious disease, Coronavirus Disease 2019 (COVID-19) has been a pandemic around the world with more than tens of millions of infected cases. The fact of heavy clinical overload of COVID-19 for medical facilities without effective treatments in most countries means that well-designed clinical trials would be extremely difficult for concurrent clinical settings. In this case, EMRs become one of the most valuable data sources for clinical studies, which place clinical BioNER as an urgent research task. However, although various benchmark corpus and text mining studies were performed on biomedical literatures, there are few BioNER studies on COVID-19 EMRs. In existing clinical studies of COVID-19, researchers extracted structured patient information either manually or from databases [10, 11]. These methods are time-consuming and laborious, while the structured information in databases lacks detailed symptoms of patients. Studies have shown that accurate symptom information is important for screening and analysis of COVID-19 [12, 13]. Most COVID-19 patients not only have respiratory symptoms such as fever, cough, and shortness of breath but also have digestive symptoms such as anorexia and diarrhea [14, 15]. At present, due to the small number of structured COVID-19 EMRs, these findings are based on small datasets and need to be further studied by a large sample. Therefore, it is urgent to find a method that can automatically extract symptom phenotypes associated with COVID-19 from a large scale of EMR data.

Traditionally, most BioNER methods are based on coarse-grained datasets, so that when extracting clinical information, there is no distinction between negated symptoms (NS) and presented symptoms (PS) [16, 17]. As a matter of fact, symptoms are subjective indications of disease, and accurately extracting symptoms and their corresponding duration is particularity important for clinical analysis [18]. Therefore, the main objective of this paper is to extract NS and PS in Chinese EMRs, respectively. For example, there is a sentence in EMRs: “The patient developed fever and cough 9 days age, without chest tightness, chest pain or other discomfort.” Among them, “fever” and “cough” are PS, which means that the patient does indeed have these symptoms, and “without chest tightness, chest pain or other discomfort” is a chunk with NS, means the patient does not suffer from both “chest tightness” and “chest pain.” If only symptom-level BioNER was performed, “fever,” “cough,” “chest tightness,” and “chest pain” would be extracted and fed to subsequent clinical analysis with errors. Therefore, the fine-grained BioNER is very important to obtain a profile of patient with precise structured phenotypes in clinical text data analysis. Moreover, some clinical studies of COVID-19 also verified our viewpoint [19–21]. Fang et al. have shown that hypertension or diabetes would lead to deterioration of COVID-19 [19]. Taken together, it is significant to avoid identifying the symptoms and diseases which patients denied, such as fever, diarrhea, diabetes, and hypertension, as their medical histories.

In this paper, we developed Human-machine Cooperative Phenotypic Spectrum Annotation System (http://www.tcmai.org, HCPSAS), constructed a fine-grained Chinese clinical corpus, and proposed phenotypic named entity recognition method (Phenonizer) for Chinese clinical IE. In our study, Chinese BioNER task was regarded as a character-level sequence labelling task to avoid the error caused by word segmentation, and contextual features were utilized to help recognize clinical named entities. More specifically, we obtained word representations of Chinese characters containing global contextual information and then fed them into the following BiLSTM layer to capture local contextual features. Finally, the dependencies of adjacent labels were captured by using the conditional random field (CRF) to determine the optimal label sequences. Computational results on the COVID-19 dataset show that Phenonizer significantly outperforms character embedding-based methods and hardly increases training time. In addition, we found that fine-grained dataset improved the performance of our method, and models trained on the fine-grained dataset are able to avoid confusing NS and PS. Finally, Phenonizer has excellent generalization ability to extract clinical information from Chinese EMRs quickly and accurately in the event of new disease outbreak.

The main contributions of our work can be summarized as follows:

- We developed a system named HCPSAS that greatly reduces the workload of annotators in the way of human-machine collaborative annotation
- Through HCPSAS, we constructed a fine-grained corpus that distinguishes NS from PS, and models trained by this corpus can avoid the error caused by symptom confusion to clinical analysis. Our fine-grained datasets can improve the performance of our model
- We proposed a recurrent neural network with bidirectional transformers for Chinese BioNER. It is the first time that deep learning has been used to extract symptoms and their corresponding duration from COVID-19 EMRs. Experimental results on the COVID-19 dataset demonstrate that Phenonizer achieves a highly competitive performance compared with Word2Vec-based methods

## 2. Related Work

Due to the practical significance of BioNER, a lot of solution approaches had been proposed. These approaches are divided into four categories: rule-based, dictionary-based, machine learning, and deep learning.

### 2.1. Rule-Based and Dictionary-Based Methods

Early BioNER systems usually rely on heuristic handcrafted rules by experts, including contextual information, grammatical constraints, synonym association, and keyword matching [22, 23]. Dictionary-based approaches employ expert-approved vocabularies to recognize entities. They are widely used because of their simplicity and performance. Most of the existing entities can be correctly identified by dictionary matching [24, 25]. However, these approaches rely heavily on manual rule bases and dictionaries. As datasets get updated, it takes a lot of manpower to maintain the rules and dictionaries, which is not an easy way. In particular, there are a large number of synonyms and ambiguous boundaries in Chinese, which bring challenges to these approaches.

### 2.2. Machine Learning-Based Methods

Machine learning-based methods usually consider BioNER as a sequence labelling task whose goal is to find the best label sequence for a given input sentence [26]. Typical methods include hidden Markov models (HMM) [27], CRF [28], and support vector machines (SVM) [29].

CRF is an undirected statistical graph model whose special case is a linear chain corresponding to a conditionally trained finite state machine. It is widely used in computer vision, shallow layer analysis, and BioNER. Its mathematical model can be described as follows: *x* represents the random variable on the data sequence to be labelled, and *y* represents the random tag on the corresponding tag sequence. In an undirected graph, *G* = (*V*, *E*), a node *v* ∈ *V* corresponding to random variable *y*_*v*_ in *V*. (*y*, *x*) is a conditional random field in which every random variable *y*_*v*_ is subject to Markov properties (*p*(*y*_*v*_ | *x*, *y*_*w*_, *w* ≠ *v*) = *p*(*y*_*v*_ | *x*, *y*_*w*_, *w* − *v*)). The conditional probability *p*(*y* | *x*) is a probability of a particular label sequence *y* for a given observation sequence *x* and can be defined as the normalized product of potential functions. The transfer characteristic function of potential function is

(1) $$\begin{array}{c} \exp\left(\underset{j}{\sum }\lambda_{j}t_{j}\left({\text{y}}_{i-1},y_{1},x,i\right)+\underset{k}{\sum }\mu_{k}s_{k}\left(y_{1},x,i\right)\right) \end{array}$$

where *t*_*j*_(*y*_*i*−1_, *y*_*i*_, *x*, *i*) is the transfer feature function of the observation sequence, namely, the labels of position *i* and *i* − 1 in the tag sequence. *s*_*k*_(*y*_*i*_, *x*, *i*) is the label of position *i* and the state characteristic function of the observation sequence; *λ*_*j*_ and *μ*_*k*_ are the hyperparameters. A set of real values of the observed values *g*(*x*, *i*) can be defined as a characteristic function to describe some characteristics of the empirical distribution of training data. When current state (in the case of a state function) or previous state and current state (in the case of a transition function) have specific values, the value of the eigenfunction will be 1. The state function *s*(*y*_*i*−1_, *y*_*i*_, *x*, *i*) and the transfer function *t*(*y*_*i*−1_, *y*_*i*_, *x*, *i*) can be expressed by *f*_*i*_(*y*_*i*−1_, *y*_*i*_, *x*, *i*); *F*_*j*_(*y*, *x*) can be defined as

(2) $$\begin{array}{c} F_{j}\left(y,x\right)=\overset{n}{\underset{i=1}{\sum }}f_{i}\left(y_{i-1},y_{i},x,i\right). \end{array}$$

By the function *F*_*j*_(*y*, *x*), the probability of observing the tag sequence *y* on the sequence *x* can be expressed as

(3) $$\begin{array}{c} p\left(y\;|\;x,\lambda \right)=\frac{1}{Z\left(x\right)}\exp\left(\underset{j}{\sum }\lambda_{j}F_{j}\left(y,x\right)\right). \end{array}$$

The main advantage of CRF is its conditional feature, which relaxes the assumption of independence required by HMM. In addition, CRF is a sequence labelling and segmentation model for discriminating training. It combines past and future observation of arbitrary overlap and aggregation. The CRF method benefits from effective training and decoding parameter estimation based on dynamic programming to ensure the existence of a global optimal solution. Nevertheless, machine learning approaches rely on predefined features and require high cost to find the best set of features.

### 2.3. Deep Learning-Based Methods

Deep learning-based methods achieve state-of-the-art performance over traditional machine learning methods in the BioNER task [7, 30]. Long short-term memory (LSTM) [31] and gated recurrent units (GRU) [32], which are recurrent neural networks (RNNs) with gated recurrent cells, can capture long dependencies in sentences. On top of these, a CRF layer is added to ensure that output label sequences are regular.

BiLSTM is a classic sequence labelling model that effectively utilizes both past information (through forward state) and future information (through backward state). For BiLSTM, given a sentence, the model predicts the label corresponding to each input character in the sentence. First, the sentence is represented by an embedding layer as a sequence of vector *X* = (*x*_1_, *x*_2_, ⋯, *x*_*n*_), where *n* is the length of sequence. Then, taking the embedded information as input to BiLSTM layer, the forward LSTM calculates forward representation $\overset{⟶}{h_{t}}$, while the other reverse LSTM calculates the backward representation $\overset{⟵}{h_{t}}$ of same sequence. The two different networks use different parameters; the hidden representation $h_{t}=\left[\overset{⟶}{h_{t}};\overset{⟵}{h_{t}}\right]$ of the character is obtained by linking its left and right context representations. Moreover, the tanh layer on top of BiLSTM is used to predict confidence score of each character's possible label as the network output score, wherein the weight matrix *W*_*e*_ is the model parameter to be learned in training.

(4) $$\begin{array}{c} {\text{e}}_{t}=\tanh\left(W_{e}h_{t}\right). \end{array}$$

For Chinese named entity recognition, Zhang and Yang [33] investigate a lattice-structured LSTM model which can effectively use Chinese word information. Recently, Devlin et al. [34] proposed a pretrained bidirectional transformer and excelled in many NLP tasks. However, this method is based on enormous computation data and computing power.

## 3. Materials and Methods

**Figure 1:**
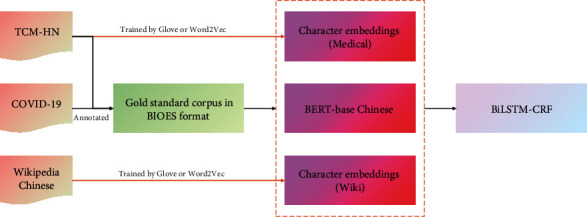
A workflow for clinical phenotypic symptom extraction in Chinese EMRs. We trained character embeddings with GloVe and W2V as baseline models. The data of TCM-HN and COVID-19 were annotated as gold standard corpus. All evaluation results were generated on test sets.

An overall workflow for our study is given in Figure 1. For clinical phenotypic symptom extraction, three methods in total based on character level were implemented and evaluated. Datasets have been processed into BIOES format. Character embeddings (GloVe_Wiki_, GloVe_Medical_, W2V_Wiki_, and W2V_Medical_) were trained by GloVe and Word2Vec (W2V) using data from Chinese Wikipedia and Henan Province Hospital of TCM. In addition, BERT-base_Chinese_ is a pretrained model, which is officially provided by Google and trained based on Chinese encyclopedia. After that, BiLSTM was used to encode the local contextual features of each word, and CRF was employed to obtain the optimal label sequences.

### 3.1. Datasets

**Figure 2:**
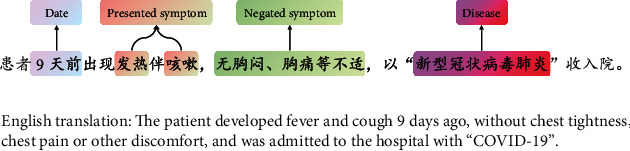
An example of annotated sentence from the COVID-19 dataset.

Our study is driven by EMRs written in Chinese. There are four datasets from Chinese Wikipedia, Henan Province Hospital of TCM, and Hubei Province Hospital of TCM. Among them, the Wikipedia data is unannotated, and the others are annotated with fine-grained rules by annotators through HCPSAS. All patient identifiers related to privacy issues had been removed before annotation.  Figure 2 shows an example of annotated sentence in the COVID-19 dataset. A detailed description of each dataset is given below.

1. The Chinese Wikipedia corpus: the first dataset is collected from Chinese Wikipedia, which has not been annotated. There are 3,745,841 sentences of 337,063,331 words with a vocabulary size of 14,261 (see Table 1). The data was trained by GloVe and Word2Vec for GloVe_Wiki_ and W2V_Wiki_
2. Chinese EMRs from Henan Province Hospital of TCM (TCM-HN): we collected EMRs from the respiratory department of Henan Province Hospital of TCM, which were mainly related to chronic lung disease (e.g., chronic obstructive pulmonary disease and asthma). It consists of the history of present illness of 41,703 patients and contains 155,566 sentences of 14,009,494 words with a vocabulary size of 3,008. On the one hand, the data was used for GloVe and W2V training to form word representations in the medical field; on the other hand, the data was used as a benchmark dataset for subsequent model training
3. Chinese EMRs of COVID-19: the COVID-19 dataset contains the chief complaint, history of present illness, current manifestations, and past history of 5,654 COVID-19 patients (including both confirmed and suspected inpatient cases) from 10 hospitals (e.g., Hubei Provincial Hospital of Traditional Chinese Medicine) in Hubei Province, China, whose amount is about 12% of TCM-HN's.  Table 2 lists the annotated entities in datasets, consisting mainly of NS and PS. We used COVID-19 data for training and evaluation of subsequent models, and all evaluations are based on entity-level exact matches
4. Chinese EMRs from Hubei Province Hospital of TCM (TCM-HB): TCM-HB dataset is composed of EMRs of fatty liver from Hubei Province Hospital of TCM, including the admission and discharge information of patients. The dataset also contains a large number of phenotypic entities such as NS and PS (Table 2), which is used to verify the generalization ability of deep learning-based methods on heterogenous data

### 3.2. Human-Machine Cooperative Phenotypic Spectrum Annotation System

**Figure 3:**
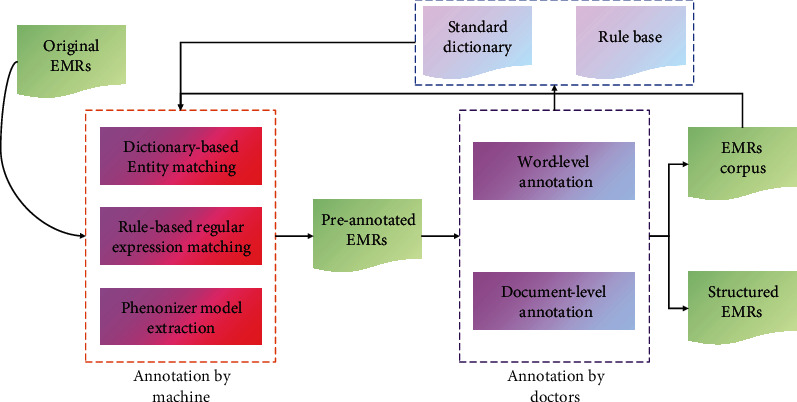
An overview of HCPSAS (http://www.tcmai.org). Our annotation system adopts human-machine collaborative annotation, in which the machine annotation includes dictionary-based entity matching, rule-based regular expression matching, and Phenonizer model recognition, and the manual annotation includes word-level annotation and document-level annotation. The iterative dictionary and rule base include standard dictionary and rule base, both of which are derived from annotation. The EMR corpus is regarded as datasets for our methods, and the structured EMRs are used for clinical analysis tasks such as patient subgroup and symptom cluster.

In order to quickly and accurately annotate row corpora, we developed HCPSAS. In this system, phenotypic entities in EMRs will be extracted in the way of human-machine collaborative annotation (Figure 3). Before original EMRs are manually annotated, it will go through the steps of machine annotation, which is driven by an iterative dictionary and rule base with preliminary seed records, to form preannotation texts. There are three parts in the machine annotation, which are dictionary-based entity matching, rule-based regular expression matching, and Phenonizer model recognition, respectively. The two former methods try to ensure the accuracy of automatic extraction in the annotating process. The latter extracts entities through the semantics in the sentences and supplements the results of the two former methods. Machine annotation is aimed at extracting most of the entities in the unannotated texts and greatly reducing the workload of manual annotation. Afterwards, clinical staff manually annotate and review preannotated texts to obtain structured EMRs and clinical corpus in a short time.

### 3.3. Architecture of Phenonizer

**Figure 4:**
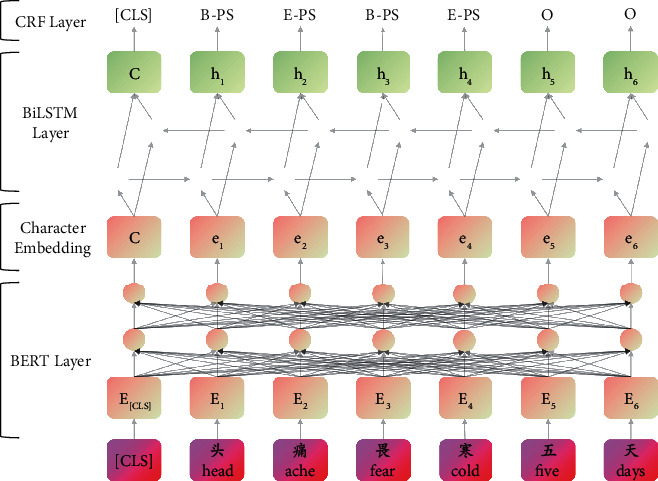
The overall framework of Phenonizer. Because of BERT, the special symbol [CLS] needs to be added before every sentence for classification output. *E*_*i*_ represents the input embedding, which is the sum of token embedding, segmentation embedding, and position embedding. *e*_*i*_ represents the contextual representation of token *ⅈ* and will be used as the input of BiLSTM. *h*_*i*_ are decoded by the CRF layer to get the optimal annotation sequences.

In this paper, we proposed a BioNER framework Phenonizer for Chinese EMRs with deep neural network (Figure 4). The model is composed of three parts, BERT, BiLSTM, and CRF. The character embeddings from BERT are regarded as the input of BiLSTM layer, and a CRF layer is added to the end of BiLSTM for decoding. In this section, the Phenonizer's architecture is described in detail following the order from inputs to outputs, layer by layer.

### 3.4. Character Embeddings from BERT

Text is a high-level abstract entity generated in human cognition. In the field of NLP, it needs to be converted into data types that can be understood and processed by neural network. Learning word representations from a large amount of unannotated text has long been a fundamental and important task in NLP field. While previous methods (e.g., Word2Vec [35] and GloVe [36]) focused on learning word representations independently, recent works have focused on learning word representations from context [37]. For instance, ELMo [38] uses a bidirectional language model, while OpenAI GPT [39] embeds contextual information into word embeddings by a transformer. BERT is a language representation model which uses bidirectional transformers to capture contextual information in text and overcomes the problem that previous language models cannot see future words [34].

BERT learns the characteristics of words from a large number of corpora through unsupervised learning. It has different structures. BERT-base_Chinese_, which we used in experiment, is a multilayer bidirectional transformer with the number of layers *L* = 12, the hidden layer parameter *H* = 768, and the number of self-attention heads *A* = 12. Different from ELMo, the pretraining task of BERT is not an N-gram language model prediction task, but a masked language model (MLM) and next sentence prediction (NSP) task. For MLM, similar to cloze task, the model randomly screened 15% of tokens for each input sequence and screened predictive tokens. For NSP, the input sequence splits sentence pairs with [SEQ], and only 50% of the sentence pairs are positive samples.

In this paper, we used contextual word representations obtained by BERT as input to our network. At same time, we trained character embeddings by GloVe and W2V and compare it with BERT to demonstrate the power of contextual word representations. For character embedding, we trained GloVe_Wiki_, W2V_Wiki_, GloVe_Medical_, and W2V_Medical_ using Chinese Wikipedia and TCM-HN datasets. We experimented with them separately and compared the results, hoping to expound that training embeddings with specialized biomedical corpora can achieve effective improvement in the BioNER domain.

### 3.5. BiLSTM Layer Using Character Embeddings

LSTM is the most commonly used model for sequential annotation tasks, which is a variant of RNNs. RNNs can continuously operate the information to ensure that the information persists, thereby solving the problem of information forgetting [31, 40]. However, in the case of long sequences, RNNs cannot handle long-term dependencies well. Therefore, LSTM came into being to address this issue. LSTM and RNNs are almost identical, except that the hidden layer updates are replaced by purpose-built memory cells to exploit long-term dependencies in sentences. In state *t*, the LSTM network takes *e*_*t*_, *C*_*t*−1_, and *h*_*t*_ as inputs and calculates its output by the following formula, where *σ* and tanh are sigmoid and hyperbolic tangent activation functions, respectively.  *i*_*t*,_, *f*_*t*_, and *o*_*t*_ represent input gate, forget gate, and output fate, respectively, and *C*_*t*_ is the storage area of LSTM unit. *W*_*j*_, *j* ∈ {*f*, *i*, *C*, *o*} are the trainable parameters of the model.

(5) $$\begin{array}{c} f_{t}=\sigma \left(W_{f}\cdot \left[h_{t-1},e_{t}\right]\right.+b_{f} \end{array}$$

(6) $$\begin{array}{c} i_{t}=\sigma \left(W_{i}∙\left[h_{t-1},e_{t}\right]\right.+b_{i}, \end{array}$$

(7) $$\begin{array}{c} {\tilde{C}}_{t}=\tanh\left(W_{C}∙\left[h_{t-1},e_{t}\right]\right.+b_{C}, \end{array}$$

(8) $$\begin{array}{c} C_{t}=f_{t}*C_{t-1}+i_{t}*{\tilde{C}}_{t}, \end{array}$$

(9) $$\begin{array}{c} o_{t}=\sigma \left(W_{o}∙\left[h_{t-1},e_{t}\right]\right.+b_{o}, \end{array}$$

(10) $$\begin{array}{c} h=o_{t}*\tanh\left(C_{t}\right). \end{array}$$

The BiLSTM was employed on embedding sequence *e*_1_, *e*_2_, ⋯, *e*_*n*_; *e*_*i*_ denotes character embedding of *c*_*i*_ by BERT, where *c*_*i*_ is a character in text sequence *c*_1_, *c*_2_, ⋯, *c*_*n*_. BiLSTM was applied to obtain $\underset{h_{1}}{⟶},\underset{h_{2}}{⟶},\cdots ,\underset{h_{n}}{⟶}\;$ and $\underset{h_{1}}{⟵},\underset{h_{2}}{⟵},\cdots ,\underset{h_{n}}{⟵}$ in the left-to-right and right-to-left directions, respectively. The hidden representation of each character is defined by

(11) $$\begin{array}{c} h_{i}=\left[\begin{array}{c} \underset{h_{i}}{⟶}\\ \underset{h_{i}}{⟵} \end{array}\right]. \end{array}$$

Next, a CRF layer was used on *h*_1_, *h*_2_, ⋯, *h*_*n*_ for sequence labelling.

### 3.6. Last Layer Based on CRF

To predict labels, BERT fed hidden representation into a classification layer, which is a simple and effective strategy when labels are independent [34]. However, entities usually consist of several words, meaning that labels do have correlations with their neighbors. For example, in CoNLL-2003 annotation [41], I-ORG (inside of the ORG) cannot follow B-PER (beginning of the PER) or O (outside of entities). Therefore, CRF was proposed to avoid false choices by adding some constraints.

For a sentence with *n* words, define *h*_*i*_ as the hidden representation of the *i*_th_ token in the sentence, *h* = {*h*_1_, *h*_2_, ⋯, *h*_*n*_} is the vector sequence of sentence, while *y* = {*y*_1_, *y*_2_, ⋯, *y*_*n*_} is the label sequence of *h*, and *Y*(*h*) is the set of all possible label sequences. Loss function was defined as

(12) $$\begin{array}{c} \text{Loss}=-\log\frac{{\text{ⅇ}}^{S_{\text{RealSeq}}}}{{\text{ⅇ}}^{S_{1}}+{\text{ⅇ}}^{S_{2}}+\cdots +{\text{ⅇ}}^{S_{N}}}=-\left(S^{\text{RealSeq}}-\log\left({\text{ⅇ}}^{S_{1}}+{\text{ⅇ}}^{S_{2}}+\cdots +{\text{ⅇ}}^{S_{N}}\right)\right)=-\left(\overset{N}{\underset{i=1}{\sum }}h_{i,y_{i}}+\overset{N-1}{\underset{i=1}{\sum }}t_{y_{i},y_{i+1}}-\log\left({\text{ⅇ}}^{S_{1}}+{\text{ⅇ}}^{S_{2}}+\cdots +{\text{ⅇ}}^{S_{N}}\right)\right). \end{array}$$

During the training process, the parameters were updated to keep decreasing the loss iteratively. There are total *N* possible sequences in *Y*(*h*), and *S*_*i*_ represents the score of sequence *i*. *S*_*i*_ is the sum of emission score and transition score. *h*_*i*,*y*_*i*__ corresponds to the score of the *i*_th_ token being labelled *y*_*i*_, which is obtained from BiLSTM. *T* denotes the matrix of transition scores in which *t*_*p*,*q*_  represents the score from tag *p* to *q*. Therefore, the sequence with the largest score is going to be given by

(13) $$\begin{array}{c} y^{*}=\text{argmax }s\left(h,\bar{y}\right),y\epsilon Y\left(h\right). \end{array}$$

In the CRF layer, the Viterbi algorithm was used to solve the optimization problem and get the result efficiently.

### 3.7. Symptom Extraction in Different Granularity

The extraction of NS and PS is the focus of this paper. So far, most of existing researches pay attention to coarse-grained symptoms. In this section, we constructed datasets that distinguish between NS and PS and those that do not, aimed at the problem that general methods only identify symptom-level entities, thus misleading clinical analysis. The two datasets as the Nonnegation (NonNeg) and With-negation (WithNeg) datasets, where the NonNeg dataset is the symptom-level dataset and the WithNeg dataset is the one that differentiates NS and PS. We trained models separately on these two datasets with the same parameters. First, we compared the symptom extraction results of two models on their respective datasets to explore whether the performance of the model in symptom extraction improved after distinguishing datasets. Secondly, we evaluated symptom-level models on the WithNeg dataset. At evaluation, we treated all recognized symptoms as PS (as did general BioNER methods). Thus, when testing, degraded models should identify the chunks containing NS in the WithNeg dataset as PS.

### 3.8. Evaluation Metrics

To evaluate the performance of BioNER methods, we selected precision, recall, and F1-score as experiment metrics:

(14) $$\begin{array}{c} \text{Precision}=\frac{\text{TP}}{\text{TP}+\text{FP}}, \end{array}$$

(15) $$\begin{array}{c} \text{Recall}=\frac{\text{TP}}{\text{TP}+\text{FN}}, \end{array}$$

(16) $$\begin{array}{c} \text{F}1‐\text{score}=\frac{2*\text{precision}*\text{recall}}{\text{precision}+\text{recall}}. \end{array}$$

True Positive (TP) is the number of entities which are identified correctly. False Positive (FP) represents the number of chunks identified as entities mistakenly. False Negative (FN) represents the number of entities that are not recognized by models. Precision is the fraction of relevant entities among the retrieved entities, while recall represents the percentage of relevant entities that are retrieved by models. F1-score is the harmonic mean of precision and recall.

## 4. Results and Discussion

In this section, we showed the experiments results with different methods independently. First, we reduced the manual labelling effort by 80% through HCPSAS. Secondly, we compared Phenonizer with different baseline models and demonstrated the strong phenotypic entity extraction ability of Phenonizer. In addition, we demonstrated that fine-grained datasets can improve phenotypic entity tasks and distinguish between NS and PS through different granularity datasets. Finally, we evaluated the performance of each method on different datasets and performed ablation experiments to demonstrate the stability of Phenonizer.

### 4.1. Human-Machine Cooperative Annotation

The three benchmark datasets used in this article were all built using HCPSAS, and the results are shown in Table 3. The number of samples of datasets ranged from 6,000 to 30,000, and the entities annotated reached the order of hundreds of thousands, but 80% of entities were annotated by machine, which achieved the original intention of human-machine collaboration in data tagging.

### 4.2. Phenonizer for COVID-19 EMRs

We trained Phenonizer models and baselines with the COVID-19 dataset, which was divided into training set and test set in a ratio of 3 : 1. Among them, one-fifth of the training set serves as development set [42]. For character embedding training, we set window size and the dimension of word embeddings to 5 and 300. In the process, we used a learning rate of 0.0001 and set hidden layer size and batch size to 128 and 8. The training for three models requires 100 epochs to accomplish and less than 10 hours at most.

The performance of different models was shown (Table 4). Comparing character embedding in general and medical domain, we found that the character embeddings obtained by training GloVe and W2V with data from biomedical field can improve the performance by about 0.001. However, it can be found that BERT's contextual representation ability makes Phenonizer better in results than W2V_Medical_ trained by biomedical field data. Compared with W2V_Wiki_, which is also trained in Chinese encyclopedia data, Phenonizer improved 0.0098, 0.0346, and 0.0226 in precision, recall, and F1-score, respectively. Therefore, the contextual information contained in BERT can promote the performance of phenotypic entity recognition with F1-score generally reaching over 0.8.

### 4.3. Comparison between Normal and Degraded Models

The model parameters in this part are the same as those in the previous experiment. By comparing the results of symptom extraction between normal and degraded models (Tables 4 and 5), it was found that the performance of symptom extraction in degraded models without NS-PS differentiation was about 0.005 lower than that of normal models, indicating that the fine-grained dataset could bring performance improvement to models.

On the other hand, we tested degraded models on the WithNeg dataset and evaluated their ability in symptom extraction (Table 6). As we envisioned, the recall of degraded models remained almost unchanged, but precision dropped significantly. The recall in results is above 0.9, indicating that most PS had been correctly recognized by degraded models. Precision dropped to about 0.6. This is because degraded models do not take into account the prefix or suffix of NS leading to the identification of the chunks with NS as PS, which is exactly what we do not want to see.

In the WithNeg dataset, there are 12,115 PS and 6,196 NS. At the same time, we also counted the proportion of NS and PS in EMRs of other hospitals or other departments (such as hepatology or surgery, no further elaboration here). In fact, each EMR contains a lot of NS, if the granularity of BioNER models' recognition of symptoms only stays at the symptom level, which is meaningless for clinical analysis.

## 5. Case Study

To show the performance of our model, we take a specific clinical sentence to demonstrate the annotation results on both presented symptoms and negated symptoms of Phenonizer.  Table 7 shows a case study comparing normal and degraded Phenonizer. In the example, Phenonizer (degraded) identified “fever,” “cough,” “chest tightness,” and “chest pain” as symptoms, which is valid on its own, but it will deliver false information for subsequent data analysis if no postprocess was conducted because the patient actually did not suffer from chest tightness and chest pain. In contrast, Phenonizer (normal) identified “fever” and “cough” as PS and “without chest tightness, chest pain or other discomfort” as NS, which would help obtain the exact symptom phenotypes for patients.

Note that both Phenonizer (normal) and Phenonizer (degraded) used the same source of character embeddings (BERT-base_Chinese_) and parameters. However, Phenonizer (degraded) was trained by a symptom-level NonNeg dataset, which is degraded from the WithNeg dataset.

In addition, we found that precision of Phenonizer for drug was relatively low, at about 0.6. Therefore, we conducted an in-depth analysis of the drug prediction results. We found that Phenonizer predicted unlabelled entities in the text. For example, in the example sentence “The patient took nifedipine sustained-release tablets 20mgqd orally for a long time to reduce blood pressure, blood pressure is unknown; has a history of type 2 diabetes and has been using insulin 30 aspartate injection for a long time,” Phenonizer successfully identified “nifedipine” and “insulin” as drug entity, but annotators omitted these two entities in the labelling process. Moreover, we found similar problems in past history. Since our dataset is symptom-specific, the absence of other entities is inevitable. The performance of Phenonizer in symptom extraction is very excellent, but a few mistakes were caused by complicated description of symptoms, such as “dissolving watery stools 5 to 6 times per day,” which brings confusion to symptom extraction.

### 5.1. Generalization Performance

To estimate the generalization performance of models with respect to different datasets (homologous and heterologous data), we trained models with the data of TCM-HN as the train set and development set and COVID-19 and TCM-HB as the test set. As for data setting, the data of TCM-HN was divided into a training set and development set in a ratio of 3 : 1, and then, all COVID-19 and TCM-HB were taken as test sets. Since the data are from different hospitals, the entity labels used for annotating are slightly different. During the experiment, we selected the same labels (PS, NS) in different datasets.  Table 8 shows the migration capability of Phenonizer on COVID-19 data; precision, recall, and F1-score are 0.823, 0.8556 and 0.8389, respectively. For the heterogenous dataset, the performance of each model decreases a lot, but Phenonizer still maintains the optimal result (see Table 9). In terms of generalization performance, Phenonizer performs better than methods based on GloVe and Word2Vec, whereupon our model has a strong generalization ability to rapidly and accurately identify entities in Chinese EMRs for clinical analysis in the face of new disease in the future.

## 6. Discussion

The basic strategy of Phenonizer is to obtain the contextual representation of words by BERT and then encode and decode the information in sentences by the combination of BiLSTM and CRF, so that the model can annotate phenotypic entities in Chinese EMRs. Moreover, Phenonizer is not limited to general coarse granularity (symptom-level) but can identify and distinguish NS and PS and extract the corresponding duration of PS. In sequence structuration, PS and duration of symptoms will be retained without NS. Therefore, our study is of great significance for clinical analysis. Because BERT is modeled based on the input of char features and in order to avoid errors caused by word segmentation, we trained character embeddings by GloVe and W2V without word segmentation.

Despite the fact that our method was successfully applied to clinical datasets, the comparison between embedding_Wiki_ and embedding_Medical_ shows that pretraining using medical data can improve the performance of model. However, due to the limitation of computing power and data volume, we can only use BERT-base_Chinese_ provided by Google at present. If we can train a Chinese BioBERT like Lee et al. [43], it will bring improvement to our method.

In addition, as in all character-based approaches, our method, while avoiding suffering from word segmentation errors, has yet to explicitly leverage word and word sequence information. It was shown that encoding a sequence of input characters as well as all potential words that match a lexicon outperforms both character-based and word-based methods [33]. This suggests that combining our Phenonizer approach with domain dictionary or knowledge graph may improve performance.

Finally, a number of recent BioNER approaches based on multitask learning have emerged, suggesting that the strong performance can be achieved by only marginally adding training time through multitask learning [44]. We would expect in the future that integrating domain dictionaries and knowledge graphs into our approach with multitask learning may enhance the semantic representation of model and improve its performance.

## 7. Conclusions

Our study provides a clinical phenotype extraction tool for Chinese EMRs. We developed HCPSAS, constructed a large fine-grained annotated Chinese EMRs corpus, and implemented a deep learning approach using character embeddings. The method using BERT as features achieves that best performance with F1-score over 89% end to end, significantly outperforming the baseline methods using GloVe and Word2Vec. Furthermore, our datasets distinguish NS and PS and enable our model to identify the two kinds of symptoms independently, so as to avoid NS being identified as PS, which will adversely affect subsequent clinical analysis. Moreover, it is verified that the ability of symptom extraction of Phenonizer can be slightly improved after distinguishing datasets. Finally, we evaluated the generalization performance of our method, using TCM-HN data and COVID-19 data for training and testing, and obtained F1-score over 83%. In addition, Phenonizer maintains optimal results on the heterogenous dataset (TCM-HB). The results demonstrate the effectiveness of deep learning methods in Chinese BioNER and the necessity of constructing a fine-grained Chinese clinical corpus.

## Figures and Tables

**Table 1:** Basic information for each dataset.

Datasets	Domain	Annotated	No. of texts	No. of sentences	No. of entities	No. of words	No. of vocabularies
Wikipedia_Chinese_	General	False	—	3,745,841	—	337,063,331	14,261
TCM-HN	Clinical	True	29,636	155,566	318,337	14,009,494	3,008
COVID-19	Clinical	True	6,105	29,663	201,567	1,726,665	2,248
TCM-HB	Clinical	True	18,555	105,075	247,291	6,394,902	2,778

**Table 2:** The number of various entities in benchmark datasets.

Entity	TCM-HN	COVID-19	TCM-HB
Presented symptom	753,541	60,364	170,047
Negated symptom	469,142	31,092	126,359
Disease	55,857	—	55,783
Tongue and pulse	2,621	1,497	—
Body parts	1,042	—	—
Operation	959	—	8,540
Date	—	18,653	—
Duration of symptoms	—	9,042	23,276
Past history	—	5,796	—
Inducement	—	3,524	—
Drug	—	3,376	23,191
Frequency	—	—	16,127
Principle	—	—	11,993

**Table 3:** The annotation results of each dataset and the proportion of machine annotations.

Datasets	No. of texts	No. of entities	No. of entities annotated manually	No. of entities annotated by machine	Machine annotation proportion (%)
TCM-HN	29,636	318,337	51,925	266,412	83.69
COVID-19	6,105	201,567	39,796	161,771	80.25
TCM-HB	18,555	247,291	52,797	194,494	78.65

**Table 4:** Comparison of different models for COVID-19 EMRs.

Models	Precision	Recall	F1-score
BiLSTM-CRF	0.8777 ± 0.0069	0.8729 ± 0.0036	0.8753 ± 0.0018
GloVe_Wiki_- BiLSTM-CRF	0.8694 ± 0.0004	0.8649 ± 0.0026	0.8671 ± 0.0012
GloVe_Medical_-BiLSTM-CRF	0.8776 ± 0.0038	0.8724 ± 0.0055	0.8750 ± 0.0011
W2V_Wiki_- BiLSTM-CRF	0.8787 ± 0.0033	0.8693 ± 0.0028	0.8734 ± 0.0013
W2V_Medical_-BiLSTM-CRF	0.8876 ± 0.0019	0.8806 ± 0.0060	0.8837 ± 0.0027
BERT-CRF	0.8793 ± 0.0008	0.9019 ± 0.0024	0.8905 ± 0.0011
Phenonizer	0.8885 ± 0.0046	0.9039 ± 0.0038	0.8960 ± 0.0009

**Table 5:** Comparison of degraded models for COVID-19 EMRs.

Degraded models	Precision	Recall	F1-score
BiLSTM-CRF	0.8660	0.8515	0.8587
GloVe_Wiki_-BiLSTM-CRF	0.8620	0.8468	0.8544
GloVe_Medical_-BiLSTM-CRF	0.8691	0.8481	0.8585
W2V_Wiki_-BiLSTM-CRF	0.8605	0.8629	0.8617
W2V_Medical_-BiLSTM-CRF	0.8624	0.8701	0.8662
BERT-CRF	0.8664	0.8916	0.8788
Phenonizer	0.8767	0.8852	0.8809

**Table 6:** Comparison of symptom extraction performance for different models on the WithNeg dataset.

Models	Precision		Recall		F1-score	
	Normal	Degraded	Normal	Degraded	Normal	Degraded
BiLSTM-CRF	0.9134	0.6111	0.9090	0.9133	0.9112	0.7322
GloVe_Wiki_-BiLSTM-CRF	0.9077	0.6076	0.9085	0.9124	0.9081	0.7294
GloVe_Medical_-BiLSTM-CRF	0.9119	0.6120	0.9077	0.9127	0.9098	0.7327
W2V_Wiki_-BiLSTM-CRF	0.9363	0.6133	0.9213	0.9287	0.9287	0.7387
W2V_Medical_-BiLSTM-CRF	0.9329	0.6144	0.9281	0.9321	0.9305	0.7406
BERT-CRF	0.9261	0.6211	0.9243	0.9254	0.9252	0.7433
Phenonizer	0.9405	0.6216	0.9387	0.9398	0.9396	0.7483

**Table 7:** Examples of symptom extraction results in both models.^a^.

Sentence (truncated)	The patient developed fever and cough 9 days ago, without chest tightness, chest pain, or other discomfort
Phenonizer (normal)	… *fever* and *cough* …, **without chest tightness, chest pain, or other discomfort**
Phenonizer (degraded)	… ***fever*** and ***cough*** …, without ***chest tightness***, ***chest pain*** …

^a^Italic and bold texts represent PS and NS, respectively. Bold italic texts denote symptoms extracted by Phenonizer (degraded). In contrast, Phenonizer (normal) considered the practical significance of symptoms in Chinese EMRs and refined symptoms into NS and PS.

**Table 8:** The symptom extraction performance of models on isomorphic data (COVID-19).

Training dataset	COVID-19			TCM-HN		
Models	Precision	Recall	F1-score	Precision	Recall	F1-score
BiLSTM-CRF	0.9128	0.9127	0.9128	0.7739	0.7675	0.7707
GloVe_Wiki_-BiLSTM-CRF	0.9064	0.9116	0.9090	0.7626	0.7715	0.7670
GloVe_Medical_-BiLSTM-CRF	0.9093	0.9144	0.9113	0.7683	0.7661	0.7672
W2V_Wiki_-BiLSTM-CRF	0.9145	0.9209	0.9177	0.7994	0.8380	0.8181
W2V_Medical_-BiLSTM-CRF	0.9164	0.9201	0.9183	0.8104	0.8457	0.8275
BERT-CRF	0.9220	0.9231	0.9225	0.8056	0.8440	0.8243
BERT-BiLSTM	0.9170	0.9220	0.9195	0.8188	0.8516	0.8348
Phenonizer	0.9211	0.9264	0.9237	0.8230	0.8556	0.8389

**Table 9:** The symptom extraction performance of models on heterogenous data (TCM-HB).

Training dataset	TCM-HB			TCM-HN		
Models	Precision	Recall	F1-score	Precision	Recall	F1-score
BiLSTM-CRF	0.7682	0.7865	0.7772	0.6512	0.5865	0.6171
GloVe_Wiki_-BiLSTM-CRF	0.7701	0.7870	0.7785	0.6510	0.6097	0.6297
GloVe_Medical_-BiLSTM-CRF	0.7705	0.7957	0.7829	0.6575	0.6104	0.6331
W2V_Wiki_-BiLSTM-CRF	0.7686	0.7964	0.7822	0.6436	0.6261	0.6347
W2V_Medical_-BiLSTM-CRF	0.7734	0.7996	0.7863	0.6623	0.6139	0.6372
BERT-CRF	0.7719	0.8179	0.7943	0.6566	0.6198	0.6377
BERT-BiLSTM	0.7688	0.8145	0.7910	0.6400	0.6406	0.6403
Phenonizer	0.7727	0.8189	0.7952	0.6438	0.6446	0.6442

## Data Availability

The data used to support the findings of this study are available from the corresponding author upon request.
